# Strategies of offspring investment and dispersal in a spatially structured environment: a theoretical study using ants

**DOI:** 10.1186/s12898-016-0058-z

**Published:** 2016-02-05

**Authors:** Adam L. Cronin, Nicolas Loeuille, Thibaud Monnin

**Affiliations:** United Graduate School of Agricultural Sciences, Iwate University, 3-18-8 Ueda, Morioka, 020-8550 Japan; UMR 7618 Institute of Ecology and Environmental Sciences of Paris, Sorbonne Universités, UPMC Univ Paris 06, 7 quai St Bernard, 75 252 Paris, France

**Keywords:** Colony foundation, Colony fission, Formicidae, Competition–colonization trade-off, Evolution of dispersal, Agent-based model

## Abstract

**Background:**

Offspring investment strategies vary markedly between and within taxa, and much of this variation is thought to stem from the trade-off between offspring size and number. While producing larger offspring can increase their competitive ability, this often comes at a cost to their colonization ability. This competition–colonization trade-off (CCTO) is thought to be an important mechanism supporting coexistence of alternative strategies in a wide range of taxa. However, the relative importance of an alternative and possibly synergistic mechanism—spatial structuring of the environment—remains the topic of some debate. In this study, we explore the influence of these mechanisms on metacommunity structure using an agent-based model built around variable life-history traits. Our model combines explicit resource competition and spatial dynamics, allowing us to tease-apart the influence of, and explore the interaction between, the CCTO and the spatial structure of the environment. We test our model using two reproductive strategies which represent extremes of the CCTO and are common in ants.

**Results:**

Our simulations show that colonisers outperform competitors in environments subject to higher temporal and spatial heterogeneity and are favoured when agents mature late and invest heavily in reproduction, whereas competitors dominate in low-disturbance, high resource environments and when maintenance costs are low. Varying life-history parameters has a marked influence on coexistence conditions and yields evolutionary stable strategies for both modes of reproduction. Nonetheless, we show that these strategies can coexist over a wide range of life-history and environmental parameter values, and that coexistence can in most cases be explained by a CCTO. By explicitly considering space, we are also able to demonstrate the importance of the interaction between dispersal and landscape structure.

**Conclusions:**

The CCTO permits species employing different reproductive strategies to coexist over a wide range of life-history and environmental parameters, and is likely to be an important factor in structuring ant communities. Our consideration of space highlights the importance of dispersal, which can limit the success of low-dispersers through kin competition, and enhance coexistence conditions for different strategies in spatially structured environments.

**Electronic supplementary material:**

The online version of this article (doi:10.1186/s12898-016-0058-z) contains supplementary material, which is available to authorized users.

## Background

One of the most fundamental life-history trade-offs is that between offspring size and number: parents with finite resources can invest in fewer, larger, offspring or more numerous, smaller offspring [1–3]. Offspring survival is typically correlated with size [3–5], such that any increase in fecundity is offset by a corresponding decrease in survivability [6]. In stable environments, this is predicted to lead to selection for a single, optimal offspring size [1, 7]. However, offspring size is often highly variable [8, 9], and this is thought to be derived from spatio-temporal heterogeneity in the environment and the strength of competition [9–12]. By incorporating these factors into spatial models it becomes clear that the demographic trade-off between offspring size and number is tied to an ecological trade-off between competition and colonization abilities [3, 13].

Environmental context can thus determine whether selection is expected to favour reproductive strategies that emphasize a capacity for colonization or for competition, or else compromise between these two traits by producing offspring of variable size [4, 14, 15]. In passively dispersing organisms such as plants, colonization ability is usually greater in small offspring, because dispersal ability is usually negatively correlated with size, and releasing more numerous (and hence smaller) offspring increases the chance of encountering favourable habitats [16]. Colonization ability will often be favoured over competition ability in conditions subject to high kin competition and inbreeding, in environments subject to temporal and spatial heterogeneity [11, 17], and in fragmented habitats [3, 16, 18, 19]. However, high habitat fragmentation can result in high dispersal costs and thus select for low dispersal [e.g., marine invertebrates: 10, wolf spiders: 20, plant seeds: 21], which may in turn select for high competitive ability. Larger offspring are generally competitively superior and can have a higher establishment success, and will be favoured under conditions of high intraspecific competition [4, 9, 22] and in adverse or stressful environments [23]. For example in the colonial ascidian *Botrylloides violaecus*, larger offspring are favoured in high competition environments whereas size does not influence survival in low competition environments [22], while in salmon (*Salmo salar*) optimal egg size is negatively correlated with environmental quality [24].

The degree to which offspring size can be adaptively modified to suit the environment is largely prescribed by physiological limitations, particularly in animals. However, an alternative mechanism of reproduction that greatly relaxes this limitation is reproduction via budding, in which organisms (or animal groups) divide into two or more new entities. Relative to single seeds or individual animal offspring, these offspring can have greatly enhanced survivability, reduced latency to reproduction, and higher competitiveness, but often at the cost of reduced dispersal [25, 26]. This mode of reproduction is widespread, occurring in a broad range of plants [e.g., 27, 28], invertebrates [25, 29, 30], and in some social vertebrates [31]. Furthermore, budding need not be to the exclusion of other strategies, and may act in concert with, or as an alternative to, more dispersive strategies [26, 32]. Whether variation occurs within or between species, the marked dichotomy in traits between budding and long-range dispersal strategies (few large vs. numerous small offspring, respectively) means that they are likely to fulfil the requirements for coexistence under a competition–colonization trade-off [CCTO; 33, 34].

Ants are excellent subjects for studies of the ecology of reproductive strategies. In the majority of species, a new colony is founded by a queen acting alone (Independent Colony Foundation, or ICF). Reproductive colonies can produce many such solitary founding queens (up to several thousand depending on species) that disperse on the wing but suffer from high mortality [25]. Colonies of the fire ant (*Solenopsis invicta*), for example, produce several hundred new queens, each of which is capable of establishing a new colony, though only around 1 % are thought to succeed [35]. Alternatively, some species employ budding dispersal, known as Dependent Colony Foundation (DCF), during which the founding queen(s) is aided by workers that leave the reproductive colony and permanently join the founding queen, and together with the queen constitute the reproductive propagule [25, 36]. Army ants (*Eciton burchelli*), for example, typically produce a single daughter colony, in which around half of the colony work force is invested [37]. The continuous presence of workers lowers mortality under DCF but, because ant workers are wingless, dispersal is severely curtailed as it is necessarily on foot. Thus, these two mechanisms represent extreme positions on the CCTO spectrum. CCTOs are thought to be important in structuring ant communities [38, 39], and reviews have identified several factors purported to favour one reproductive strategy over the other, including predation pressure, habitat patchiness, resource availability, competition, nest site stability, nesting site limitation, and climate [25, 40–43]. However, very few empirical data are available regarding which environmental conditions favour which strategies and when particular strategies will dominate or coexist. Understanding ecological conditions favouring either strategy is important for making predictions regarding species interactions, performance of species under climate/habitat change, and movements of invasive species.

The CCTO gives clear expectations regarding the coexistence of dispersive and less dispersive strategies [33, 34]. Coexistence can occur provided the less dispersive strategy is sufficiently competitive, that is, excludes the dispersive strategy in local patches in the absence of migration. Strict boundaries exist to this coexistence however, as the differences in dispersal rates have to be high enough, or the probability of local extinction events large enough, to allow the maintenance of the dispersive strategy [33]. It is however important to stress that this original CCTO model is based on the occupancy of identical patches comprising a completely homogeneous space and as such does not consider spatial variation in species densities or environmental factors. In the present work, we add two components that are particularly pertinent for the selection of reproductive strategies in ants: (1) life-history parameters including dispersal; (2) a spatially explicit environment allowing environmental heterogeneity. Variation in these characteristics can influence strategy success and thus may lead to deviations from patterns expected under a more traditional CCTO-model. Indeed, previous studies have clearly stated that spatial heterogeneity of the environment plays a major role in the evolution of dispersal: spatially heterogeneous distribution of resource per se can favour the coexistence of low and high dispersal strategies [44]. Coexistence is further reinforced under temporal fluctuations in resource distribution [45, 46], a feature we explicitly tackle here, as precisely these circumstances arise from local dynamics associated with colony energetic requirements (component (1) above) in combination with a heterogeneous resource supply (component (2) above). We adopt an agent-based modelling approach in an attempt to tease apart when coexistence can be explained by the CCTO and when coexistence is linked to spatial and temporal fluctuations in the environment, and determine under which conditions of both the above strategies become exclusive. This approach allows us to also explicitly model resource competition through interactions of agents within patches, and thus contrasts with many patch models which assume instantaneous replacement. In this study we compare the performance of two reproductive strategies that vary markedly in investment and dispersal characteristics in different simulated environments, in which variations in patch quality and in local colony density associated with a disturbance process create asynchronous spatio-temporal variation in environmental constraints. We mathematically derive predictions of how competitive ability will be influenced by variation in a set of life-history and environmental parameters and test these predictions explicitly using our agent based model. We also test the following broad expectations: (1) that in a spatially homogeneous environment, coexistence rules depend on competition and colonization abilities [33], so that we expect ICF to coexist and eventually displace DCF when disturbance rates increase or when differences in competitive abilities are low, and (2) that when spatio-temporal variation in the environment is introduced, coexistence of the two strategies is facilitated, in the sense that larger ranges of parameter values allow for coexistence compared to analogous scenarios in homogeneous environments, in a manner similar to that thought to govern plant communities [e.g., 9, 47, 48].

## Methods

### Reproductive strategies in ants

We use a spatially explicit agent-based model implemented in NetLogo 5.1 [49]. Although recent works indicate that DCF can result in offspring comprising a range of sizes [25, 50], we here consider only the extreme case found in honey bees and army ants, in which the colony divides more-or-less evenly into two parts [37, 51]. The alternate strategy, ICF, is in contrast characterized by the production of numerous small offspring (individual queens). ICF and DCF differ in terms of offspring number, offspring size (hence initial offspring growth rate and maintenance cost), offspring mortality and offspring dispersal distance (Table 1). Collectively, these traits effectively define the colonization ability of each strategy [sensu 33] and help define their competitive ability (see below). We adopt an ‘all else equal’ approach, and thus assume that agents differ only with regard to the above parameters. Thus, agents of the two strategies reproduce at the same size (hence identical growth rate and maintenance cost), and invest equally in reproduction (although apportion resources differently among their offspring). Furthermore, for simplicity, we do not allow strategies to evolve and assume no gene exchange between strategies, and thus in this sense our simulations represent interactions between separate species rather than between different reproductive modes within a single species. Agents model ant colonies as a whole (i.e., as super-organisms), and hence number and size of agents refer respectively to the number and size (number of workers) of colonies. Agents were divided arbitrarily into ‘large’ (defined as those with size exceeding 75 % of their maturity threshold [=reproductive size; Table 1]) and ‘small’ to broadly investigate patterns of agent maturity.

**Table 1:** Model parameters, ranges of values tested, and reference values

Parameter	Description	Values
Reproduction (fixed parameters)		
Dispersal range	Maximum distance new propagules can disperse from the parent colony^a^	DCF: *1*; ICF: *30*
Dispersal mortality	Chance dispersing propagules die from predation or environmental hazards (% mortality)	DCF: *10*; ICF: *90*
Offspring size	Size of new propagules	DCF: half of the colony^†^; ICF: *20*
Offspring number	Number of new propagules	DCF: *1*; ICF: 1/40 colony size^b^
Life-history		
Longevity	Number of steps colonies can live for (= queen lifespan)	5; 6; 7; 8; 9; *10*; 15; 20; 25; 30; 35
Maintenance cost	Percentage of resources (= individuals) “consumed” for survival per time step	1–10 % by 1 % increments; 15 %; 20 % (*5* *%*)
Maximum growth rate	Maximum multiple by which colonies can grow each step (provided they collect sufficient resources)	×1, ×2, ×3, ×4, ×*5*, ×6, ×8, ×10, ×15, ×20, ×25
Maturity threshold	Size at which colonies reproduce	100 to 1400 workers by 100 increments (*700*)
Reproductive investment	Percentage of resources (= individuals) invested into offspring when reproducing	5 %; 10–90 % by 10 % increments; 95 % (*50* *%*)
Environmental		
Disturbance	Percentage of patches on which all agents are killed via catastrophic events each step	0, 1, 2, 3, 4, 5, 7, 10, 15, 20 and 25 % (*5* *%*)
Resources	Resources in each patch are reset at each step. Good patches receive 100 % and bad patches 50 % of the base value.	150–450 by 25 increments (*300*)
Aggregation	Spatial aggregation of patch quality based on Hurst exponents (H) in a fractal algorithm	Uniform^c^, *random*, H = 0, H = 0.5, H = 1

Fixed parameters are those that define the reproduction strategies and are unique to each. Life-history parameters were shared between strategies while environmental parameters defined the spatio-temporal environment. Values in ‘aggregation’ refer to Hurst exponents in the fractal algorithm. Reference values are shown in italics. All simulations lasted 1000 steps, which ensured equilibrium was reached at the end of the simulations, except simulations of invasion. These lasted 3000 steps with 1 colony of the invading strategy added after completion of step 500, and visual checks showed that all simulations reached equilibrium whether invasion occurred or not

Random and aggregated (H = 0, 0.5 and 1) environments had 50 % good and 50 % bad patches. Uniform environments consisted entirely of medium patches, with resources intermediate between those of good and bad patches (i.e., 75 % of a good patch in the other simulations) to maintain a constant amount of resources landscape level

^a^For each propagule, the actual distance of dispersal was drawn from a uniform distribution between 0 and the maximum distance. A uniform distribution was chosen as we have no information regarding dispersal kernels in ants or reason to expect that an alternative distribution would apply equally well to both strategies

^b^Note that offspring size and number were only partially fixed, as these factors also depended on reproductive investment for one of the two strategies in each case (see main text), and the values given assume reference levels of reproductive investment (50 %). The size of new DCF propagules was derived as (colony size × reproductive investment) whereas in the case of ICF, colonies produced (colony size × reproductive investment/20) new propagules

### Model overview

**Fig. 1:**
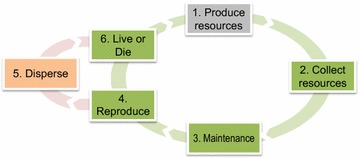
Flow diagram of one simulation step. Each step represents one reproductive cycle (i.e. one year) and consists of several phases: (*1*) resources in each patch are reset to their base value ±10 %; (*2*) the resources in each patch are divided among the agents present in the patch in direct proportion to their size. Agents then utilize this share of resources to grow. (*3*) Each agent ‘consumes’ a proportion of its workers to cover maintenance costs. (*4*) Agents larger than a threshold size produce offspring following the agent’s reproductive strategy (ICF or DCF), with the resources invested in reproduction removed from the parent agent and converted to dispersing offspring; (*5*) offspring disperse immediately after being produced following their dispersal strategy (ICF or DCF). Those surviving dispersal become new agents on the arrival patch; (*6*) agents die from old age, from starvation via maintenance costs (if they reach size 0) or from patch-level stochastic extinction (disturbance). *Colours* represent entities in the model: *light grey* represents patch actions, *green* refers to colony actions, and *orange* indicates offspring actions

A flow diagram for the Netlogo model is presented in Fig. 1, while a full ODD (Overview, Design concepts and Details [52]) is provided in Additional file 1, and the code for the model in Additional file 2. The toroid landscape of 31 × 31 patches is populated with a set number of randomly distributed agents. Newly dispersing agents (produced via reproduction of existing agents) may establish themselves in any patch within their dispersal range, but thereafter remain sessile. Agents collect resources from the patch they inhabit and each resource acquired is converted into one individual (i.e., increase colony size). They consume individuals (i.e., lose colony size) to cover maintenance costs and to produce offspring. Agents interact with other agents present in the same patch indirectly through resource competition.

### Scenarios and parameters modelled

Three scenarios were simulated. In “single-strategy” scenarios, only agents of one strategy were present. Strategies were scored as inviable if they could not survive under certain combinations of parameters. These scenarios were run independently for each strategy. In the “two-strategy” scenario the two strategies initially co-occurred and competed for resources until reaching equilibrium, allowing inference of the cost of competition between strategies when contrasted with single-strategy scenarios. Equilibrium was defined based on visual checks of the point at which populations were stable, both in terms of birth/death rates of each strategy and, in two-strategy conditions, the proportions of each strategy. The outcome of two-strategy scenarios was defined as either “coexistence” if both strategies persisted at the end of the simulation, or “competitive exclusion” if only one remained. Finally, an “invasion” scenario was modelled to determine whether each strategy, when introduced at low abundance (a single new agent), could invade the other at equilibrium. An invasion was deemed a success when one or more colonies of the introduced strategy remained at the end of the simulation, regardless of the impact on the host strategy. Invasion scenarios allowed us to determine whether the resident strategy was an evolutionary stable strategy (ESS; when there were no successful invasions for any of the replicated simulations for that parameter set) and assess the role of frequency dependence. Each simulation was replicated 50 times for each set of parameter values (see below), and data presented are the means of the end conditions for these 50 runs. Replications were required to account for inherent stochasticity in our model (see Additional file 1). Although single-strategy scenarios achieved equilibrium at <500 steps, both single and two-strategy scenarios were run for 1000 steps for comparability. Invasion scenarios were run for 3000 steps with one reproductive-size agent of the invading strategy added to a randomly selected patch at step 500. Thus the resident strategy had reached equilibrium by the time the invader was introduced, and we confirmed that the remaining 2500 steps ensured that the invader strategy also had time to reach equilibrium. Visual checks of the temporal trends confirmed that strategies were at equilibrium by the time the simulations ended in each case.

We used two main forms of environment: ‘uniform’ and ‘harlequin’ landscapes [53, 54]. In uniform landscapes, all patches were of ‘medium’ quality, whereas harlequin landscapes were constructed of ‘good’ and ‘bad’ patches in equal proportions. Bad patches had half the quality of good patches and medium patches were of intermediate quality between good and bad patches, hence the resources available at the landscape level was equal in both harlequin and uniform landscapes. Patch distribution in harlequin landscapes was either ‘random’ or ‘aggregated’. In ‘random’ landscapes, good and bad patches were randomly distributed. In aggregated habitats, fractal landscapes were generated using the midpoint-displacement method [55] and Hurst exponents of 0, 0.5 and 1, to generate low, medium and high levels of ‘spatial contagion’ of like patches [56].

We modelled agents using four fixed reproductive parameters and five variable life-history parameters (Table 1). The fixed parameters defined the different reproductive strategies, effectively making DCF a ‘competitor’ strategy and ICF a ‘colonizer’ strategy (sensu Tilman [33]; Table 1). Of the four ‘fixed’ parameters, offspring size and number were only partially fixed in that one of the two parameters also depended on reproductive investment (a variable parameter) in each case. Offspring size was fixed for ICF colonies at 20 (representing a lone queen and her fat and muscle reserves, from which the first workers are derived in ‘claustral’ ICF [57]), while the number of offspring was determined by the resources invested. DCF colonies on the other hand divided into two, thus producing a single offspring, the size of which was dependent on the level of reproductive investment and the size of the parent. Dispersal mortality was assumed to be high in ICF and low in DCF and, for simplicity, consistent in DCF propagules regardless of size (though offspring size was potentially variable). For each variable parameter, we determined a “reference value” based on available data for ants [reviewed in 25] and for *Cataglyphis cursor* in particular [50]. The three scenarios were simulated using these reference values, in order to develop a baseline using biologically realistic parameters. In addition, we modelled three environmental parameters (Table 1) in order to investigate the influence of extrinsic factors on the success of each strategy. Subsequently, we investigated the influence of each variable parameter by repeating all scenarios while varying one parameter and holding the others constant at their reference values (Table 1). We initially consider the influence of varying life-history parameters on the CCTO in a uniform environment. We subsequently contrast these patterns with an otherwise identical set of simulations performed in a harlequin landscape to elucidate the relative importance of habitat spatial structure versus the CCTO in the coexistence of the two strategies. In total, 42,250 simulations were run for 170 unique combinations of parameter values.

### Linking model parameters to competition and colonization ability

In our model, colonization ability is defined by the fixed parameters of each reproductive strategy (Table 1). As the CCTO dictates that a colonizing strategy (ICF) will dominate in the absence of sufficiently strong competition from a less-dispersing strategy (DCF), success depends on relative competitive ability. We thus momentarily set aside the spatio-temporal variation in resources to focus on the intrinsic differences in competitive ability of the two strategies. The aim of this analysis is to generate predictions of when the CCTO per se explains the dominance or coexistence of strategies. Our model relies on the acquisition and allocation of resources, and the two strategies (ICF and DCF) compete for such resources locally. Traditionally, the competitive hierarchy among species has been determined by the amount of resources remaining at equilibrium, with the most competitive species being the one that leaves the lowest amount [i.e., the R* rule, 58]. In our case, however, resources in each patch are replenished at the beginning of each time step, rendering this definition impractical. Furthermore, because competitive ability in our model is the product of multiple interacting life-history traits, it is best defined by the proportion of the lifespan spent reproducing.

Assuming that resources cover maintenance costs, the dynamics of the size of an agent follow Malthusian growth, so that:

1 $$n_{t} = n_{0} \lambda^{t}$$

where *n*_*t*_ is the size of the agent at age *t*, *n*_*0*_ is the size of the agent at birth and *λ* is the local growth rate.

Given Eq. (1), size at maturity *n*_*m*_ is reached at age *t*_*m*_ such that:

2 $$t_{m} = \frac{{log\left( {\frac{{n_{m} }}{{n_{0} }}} \right)}}{log\left( \lambda \right)}$$

Life span depends on two processes. We have defined a longevity *t*_*l*_ that sets the maximum number of steps the agent can live. However, given that disturbance occurs at rate *e*, the agent may die sooner. Since such disturbances follow a Poisson process associated with an event rate *e*, the expected time interval between disturbances is *1/e*. Considering that dispersers will arrive in a patch on average at the mid-point between two disturbances, agents have an amount of available time that is on average $\frac{1}{2e}$ to grow and reproduce. Hence, the average age of death *t*_*d*_ is the minimum value described considering both longevity and disturbance, and can be written:

3 $$t_{d} = min\left( {t_{l} ,\frac{1}{2e}} \right)$$

Competitive ability *Q* is then defined as the time spent reproducing given the generation time *t*_*d*_:

4 $$Q = \frac{{t_{d} - t_{m} }}{{t_{d} }}$$

Note that our two strategies clearly differ in their initial size *n*_*0*_, noted *n*_*icf*_ and *n*_*dcf*_ respectively. Differences of competitive ability can then be written as:

5 $$\varDelta Q = Q_{dcf} - Q_{icf} = \frac{{t_{d} - t_{m,dcf} }}{{t_{d} }} - \frac{{t_{d} - t_{m,icf} }}{{t_{d} }}$$

Using Eq. (2) and simplifying one gets:

6 $$\varDelta Q = \frac{{log\left( {\frac{{n_{m} }}{{n_{icf} }}} \right) - log\left( {\frac{{n_{m} }}{{n_{dcf} }}} \right)}}{{t_{d} log\left( \lambda \right)}} = \frac{{log\left( {\frac{{n_{dcf} }}{{n_{icf} }}} \right)}}{{t_{d} log\left( \lambda \right)}}$$

Equation (6) highlights how the parameters of the model affect the relative competitive abilities of the two strategies. First note that, because the initial size of DCF propagules is larger than the initial size of ICF propagules, DCF is always the best competitor (i.e., $\varDelta Q$ is positive). Any increase in size asymmetry inflates this competitive difference (i.e., $\varDelta Q$ increases with the ratio of offspring size $\frac{{n_{dcf} }}{{n_{icf} }}$). This influence due to initial size is entirely linked to the fact that maturity is reached sooner for DCF than for ICF strategies. Equation (6) also highlights that any increase in *t*_*d*_ (i.e., higher longevity and/or lower disturbance rates) will erode the difference in competitive abilities. Similarly, a higher growth rate within patches decreases competitive asymmetry. While the analysis above assumes that maintenance costs are covered, note that incorporating such maintenance costs lowers the realised growth rate $\lambda$, so that we expect from Eq. (6) that maintenance costs will exacerbate differences in competitive abilities.

Equation (6) therefore allows some predictions regarding the influence of the different parameters on the relative position of the two strategies along the CCTO based on relative competitive abilities alone: any increase in longevity or growth rate, or any decrease in the size asymmetry of offspring or disturbance rate, will help ICF strategies to coexist, and possibly exclude (given their advantage in terms of dispersal), DCF strategies. Maturity threshold is expected to have no effect on the competitive hierarchy as it influences the competitive ability of both strategies equally. However, the outcome of the CCTO relies not only on competition, but also on colonization constraints. For example, increased disturbance will advantage the colonizing strategy by generating increased vacancies in the landscape [33, eq. 4.1] while it only affects competitive ability (Eq. 6) under certain conditions (when *t*_*d*_ is constrained by disturbances rather than by longevity, Eq. 3). Thus, because of the differences in dispersal ability between the two strategies, we expect increased disturbance to more systematically favour ICF. Also, we expect that DCF will be favoured in environments of lower spatial heterogeneity (i.e., higher habitat aggregation) because higher clustering of good habitat can favour short-range dispersers while long-range dispersers will be unaffected. Finally, arguments presented above suggest that introducing spatially explicit environmental heterogeneity will lead to a broader range of conditions supporting coexistence of strategies. Table 2 summarizes expected outcomes of competition between strategies based on these arguments.

**Table 2:** Summary of factors favouring each strategy

Parameter	Expectations		Outcomes of simulations				
	Increase expected to favour	Basis of expectation	Single strategy		Two-strategy		Follows predictions?
			DCF	ICF	Low	High	
Life-history							
Longevity	Colonizer	Equation 6	++	+++	ICF	ICF	Y
Maintenance cost	Unclear	^a^	–	–	DCF	ICF	na
Maximum growth rate	Colonizer	Equation 6	none	+	DCF	ICF	Y
Maturity threshold	None	Equation 6	–	–	DCF	ICF	na
Reproductive investment	Unclear	^b^	+	++	DCF	ICF	na
Environ.							
Disturbance	Colonizer	Tilman	–	–	DCF	ICF	Y
Resources	Colonizer	Equation 6	+++	++	ICF	DCF	N
Aggregation	Competitor	^c^	+	–	ICF	DCF	Y

The predictions of increasing the parameter of interest, and basis for this prediction, are given in the first two columns (see main text). The single-strategy columns indicate the influence of the parameter on the abundance of each strategy, with increasing positive or negative effect indicated by increasing number of ±symbols. The dominating strategy at high and low parameter values under competition conditions is given in the two-strategy columns. The final column notes whether results followed the predictions (see “Results”). Tilman refers to Eq. 4.1 in Tilman [33]

^a^Equation 6 predicts increasing maintenance costs will exacerbate differences in competitive ability thus favouring DCF, but increasing maintenance cost will also lead to more colonization opportunities by decreasing overall occupancy (see also main text)

^b^Equation 6 predicts that DCF competitiveness is favoured by increasing asymmetry in propagule size (i.e., increasing reproductive investment) but ICF will also be advantaged through increased colonization ability from increased number of propagules [33, eq. 4.1]

^c^Higher habitat aggregation is expected to favour the competitive strategy as increasing aggregation leads to increasing probability of dispersal to good patches for the low disperser, while colonizers disperse equally to good and bad habitat irrespective of their aggregation. This is a novel characteristic of our spatially explicit model

## Results

### Effects of colonization–competition constraints on community dynamics in a uniform landscape under reference conditions

**Fig. 2:**
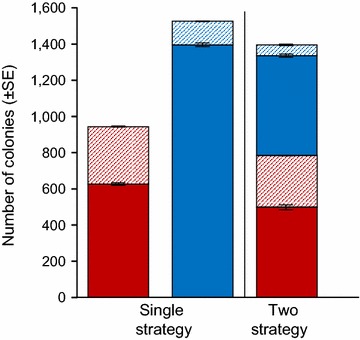
Abundance of colonies in a uniform environment. *Stacked* *bar-graphs* indicating number of colonies (mean ± SE for DCF in *red* and ICF in *blue*) for single strategy and two-strategy scenarios over 50 simulations partitioned. *Individual bars* represent the total number of colonies in each simulated environment, broken down into small colonies (*solid colours*) and large colonies (*hashed bars*). For single-strategy scenarios, data for each strategy were obtained from independent simulations. For the two-strategy scenario they are from the same set of simulations

Under single-strategy scenarios, ICF had a higher total abundance than DCF, though it produced fewer large colonies (Fig. 2) and had a lower mean colony size (Additional file 3: Figure A1). Both DCF and ICF suffered from competition between strategies, as both produced fewer colonies under the two-strategy scenario, though competition had a lower negative impact on DCF than ICF (Fig. 2). While the number of colonies was impacted by competition in both strategies, mean colony size was largely unaffected (Additional file 3: Figure A1).

### Influence of life-history parameters

**Fig. 3:**
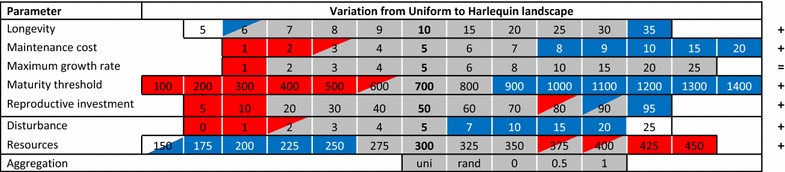
Comparative invasion matrix for uniform and harlequin landscapes. *Numbers* indicate parameter values tested, with the reference conditions for each parameter indicated in *bold* in the central column. *Background colours* indicate outcomes of invasion scenarios as either an ESS for DCF (*red*), ESS for ICF (*blue*), coexistence of both strategies (*grey*) or inviable conditions for both strategies (*white*). Panes are split to indicate outcomes in a uniform landscape (*upper left* of each pane) and a harlequin landscape (*lower right*) such that two-toned panes indicate parameter values for which different environments produced different ESS conditions. Nine changes were found when switching from uniform to harlequin landscapes: one from no strategy surviving to ICF, and the remainder from ESS to coexistence [two from ICF to coexistence (*blue* to *grey*) and six from DCF to coexistence (*red* to *grey*)]. *Symbols* on the *right hand end* of the figure indicate whether the conditions for coexistence of both strategies relative to uniform landscapes were broadened (+) or unchanged (=). For aggregation: *uni* uniform, *rand* random

Whereas both strategies could coexist under our reference values, we found conditions of competitive exclusion and ESSs for most parameters when values deviated from reference conditions (Fig. 3). For invasion scenarios, equilibrium conditions were similar to the corresponding two-strategy scenario with identical parameter values when invasion was successful, while equilibrium conditions for failed invasions were akin to single-strategy scenarios (Additional file 3: Figure A2). This indicates that the initial number of colonies had little impact on the equilibrium state of trials, suggesting the absence of alternative stable states in our simulations. Table 2 compares our expectations to the results of simulations under the range of parameter values tested, while a comparative ESS matrix is provided in Fig. 3. Figures illustrating trends over each of the parameters are given in Additional file 3.

These data indicate that our predictions based on differences in competitive ability (Eq. 6) and on differences in colonization opportunity [based on 33] are borne out in three of four cases (see Table 2): increases in longevity, maximum growth rate and disturbance all favour the colonizing strategy, presumably by reducing the asymmetry in competitive ability (Eq. 6). Our prediction that patch aggregation should favour the competitor was based on the explicit consideration of space novel to our agent-based model and is also borne out (see below).

We made no predictions for changes in maintenance cost, maturity threshold and reproductive investment because of contradictory expectations from a competition and colonization perspective. However, a closer look at the influence of these parameters in light of our results suggests that colonization effects predominate. Firstly, while increasing maintenance cost was detrimental to both strategies, the impact was greater on DCF, and as a result ICF became an ESS at high maintenance costs, although it was excluded at low values (Fig. 3; Additional file 3: Figure A3b). Although Eq. (6) predicts that higher maintenance costs will exacerbate differences in competitive abilities, our results suggest that this effect is exceeded by the influence of colonization, as increasing maintenance cost will also reduce growth and overall occupancy, leading to more colonization opportunities. Secondly, no prediction was made for the influence of maturity threshold as Eq. (6) predicts that maturing later will influence the competitive ability of both strategies equally. Simulations indicate however, that increasing maturity threshold was relatively more detrimental to DCF. This result can be explained by induced effects on fecundity. Whereas DCF agents produce a single offspring under any maturity threshold, ICF agents will generate more propagules with increasing maturity threshold (and thus size at reproduction). This increase in fecundity produces a colonization advantage, as higher maturity thresholds will also lead to fewer colonies achieving reproductive size, and thus more colonization opportunities. Finally, increasing reproductive investment has two opposing effects: (1) increasing the competitive advantage of DCF (Eq. 6) via greater offspring size asymmetry (as ICF offspring size remains the same), and (2) increasing ICF colonization ability (as ICF produces more propagules). Due to these two contradictory mechanisms, we expect the outcome to be context-dependent. Simulations suggest that the latter of these two influences is dominant for parameter values we investigated.

Finally, for variation in resources, results contradicted our predictions. Whereas Eq. (6) predicts that increasing resources erodes the competitive advantage of DCF via allowing higher growth rates, DCF was increasingly dominant with increasing resources. Two factors may contribute to this pattern: firstly, colony growth in our agent-based model is limited by both maximum growth-rate and resource availability, and these factors influence DCF and ICF differently. ICF offspring, being small, are initially growth-rate limited, as they cannot use all available resources (their growth is limited to size × maximum growth rate), whereas DCF colonies are only ever resource limited, and are thus immediately advantaged by any increase in resources. ICF colonies are advantaged only once they are large enough to exploit all the available resources in a patch (Additional file 3: Figure A5). This analysis is also supported by the effect of maximum growth rate in single strategy conditions: while there was no effect on the abundance of DCF, maximum growth rate had a strong influence on the abundance of ICF, but only at lower values (i.e., until ICF is no longer growth-rate limited; Additional file 3: Figure A3c). Secondly, low dispersal of DCF colonies means that they often disperse within the patch occupied by their parent, and thus face a higher probability of kin competition. Because this bias is higher in less saturated environments, its effect becomes attenuated with increasing resources (Additional file 3; Figure A6).

### Abundance of strategies in a harlequin landscape under reference conditions

**Fig. 4:**
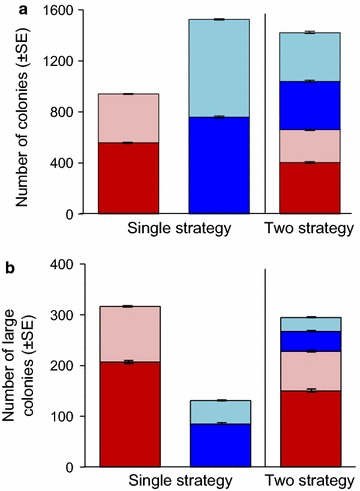
Abundance of colonies under in a harlequin environment under reference parameters and with random distribution of patches. *Stacked bar graphs* indicating numbers of colonies for single strategy and two-strategy scenarios. *Individual bars* represent the total number of colonies in a given simulated environment (mean ± SE for DCF in *red* and ICF in *blue*) partitioned into those present on good (*dark*) and poor (*light*) patches. **a** Shows all colonies, while **b** shows only large colonies

The heterogeneity in resources between good and bad patches in harlequin landscapes led to a distribution bias: when the two strategies were in competition, DCF was more abundant on good patches (61.1 %), whereas ICF was equally abundant on good (49.9 %) and bad patches. This difference was also found under single-strategy scenarios (DCF = 59.3 % in good patches; ICF = 49.8 %; Fig. 4), suggesting the pattern stems from differences in dispersal ability rather than competitive exclusion of ICF from good patches. Indeed, since colonies on good patches have higher productivity than those on bad patches and DCF offspring have restricted dispersal, DCF can be expected to become aggregated on good patches (particularly in aggregated landscapes—see below). In contrast, ICF offspring disperse throughout the environment and thus establish themselves equally often on good and bad patches. However, the distribution of large colonies suggests that DCF also excludes ICF from good patches to some extent. The single-strategy scenarios show that large colonies of both DCF and ICF were more abundant on good than on bad patches (65.3 and 64.7 %, respectively), which is expected as the growth rate is higher on good patches, and while this percentage remained the same for DCF in the two-strategy scenario (65.6 %) there was a moderate decrease for ICF (58.9 %, Fig. 4). This indicates that the distribution of the two strategies at equilibrium in a harlequin landscape is a product of both relative dispersal and competitive ability.

### Influence of habitat aggregation

**Fig. 5:**
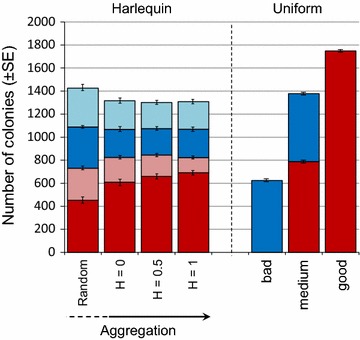
Influence of habitat aggregation and resources on strategy abundance. *Stacked bar-graphs* indicating total number of colonies in each simulated environment (mean ± SE for DCF in *red* and ICF in *blue*) for two-strategy scenarios in landscapes of varied spatial heterogeneity (*left*) or varied resources (*right*). *Left hand bars* show influence of habitat aggregation on abundance of strategies in harlequin landscapes divided into colonies on good (*dark*) and bad (*light*) patches under the reference parameters. Note that the degree of habitat aggregation is not contiguous between random and aggregated landscapes (H = 0–1). *Right hand bars* illustrate the dominance of DCF in an ‘all good’ landscape versus and ‘all bad’ landscape compared to our reference uniform conditions

DCF was able to exploit more good-habitat in aggregated versus random harlequin environments (Fig. 5), though no ESS conditions were found in invasion simulations for the levels of aggregation explored (Fig. 3). Similar patterns were found for single strategy scenarios (Additional file 3). This result can be explained by the fact that DCF, having local dispersal and propagules produced mainly on good patches, has a higher probability of dispersing to other good patches when the landscape is aggregated. The same is not true for the long-range disperser (ICF) which reaches patches of both kinds with equal probability. Interestingly, the overall abundance of strategies under reference conditions was very similar for harlequin landscapes and uniform landscapes (composed entirely of medium patches). This shows that varying spatial heterogeneity while holding resources constant has little effect on overall abundance, and only a subtle effect on relative success (favouring the colonizer in more heterogeneous environments). In contrast, abundance differed markedly when resources differed (i.e., harlequin and uniform landscapes with medium resources versus environments of all good or all bad patches, Fig. 5). This is because increasing resources raises carrying capacity while simultaneously influencing the relative success of the two strategies and favouring the competitor (conversely, decreasing resources lowers carrying capacity and favours the colonizer). This suggests an outstanding influence of resource availability (a competitive ability effect) over landscape structure (a colonization ability effect).

### Influence of spatial heterogeneity on coexistence conditions

As for uniform landscapes, the two strategies co-occurred in harlequin landscapes under a wide range of parameter values. Patterns of competitive exclusion and ESS conditions were very similar to that observed in uniform landscapes (Additional file 3: Figure A4). However, a notable difference is that in line with predictions, coexistence (i.e., mutual invasibility) occurred over a broader range of parameter values for four of the five life-history parameters and both of the comparable environmental parameters (Fig. 3), so that including spatial variation in the environment enhances the maintenance of diversity overall. This result conforms to our expectations, as spatial heterogeneities provide more opportunity for niche partitioning of species in space [59], thereby favouring coexistence.

## Discussion

Organisms differ markedly in the pattern of reproductive allocation among offspring and this can have strong implications for offspring competitive and colonization ability. Our agent-based model demonstrates that simulated organisms (here ant colonies) employing markedly different reproductive strategies (colonizer vs. competitor) can coexist under a broad range of life-history and environmental parameter values in a uniform environment. While a range of mechanisms can give rise to species coexistence in meta-communities [reviewed in 59], we proposed that the asymmetry in life-history traits that defines colonizer and competitor strategies could lead to the coexistence of species employing alternative reproductive strategies via a CCTO. In support of this, results for variation in two of our five life-history parameters (i.e., where predictions were possible) were consistent with the predictions of our competitive ability model, while the patterns observed in the remaining three parameters (where predictions were unclear or null) were consistent with an influence of these parameters on colonization ability following the CCTO model of Tilman [33]. While several studies have suggested that the assumptions of the CCTO are not biologically realistic and often violated [9, 60, 61], ant reproductive strategies may suit this model quite well, particularly the two extreme forms characterized by DCF (large, competitively superior offspring with limited dispersal) and ICF (numerous, vulnerable offspring with high dispersal).

Our model diverges from a typical CCTO-model in two important ways: (1) we consider variation in patch quality and (2) we model dispersal distance, whereas Tilman’s [33] model considers a homogeneous space and global dispersal. Considering dispersal explicitly is an important distinction, as evidenced by the different distribution of strategies among good and bad patches in harlequin environments. This is because colonization ability depends not only on the number of offspring produced, but also their ability to disperse and the spatial structure of the environment, and these factors may have differing effects [e.g., 9, 60, 62, 63]. Furthermore, allowing within-patch dynamics rather than assuming instantaneous replacement means that once a colonizer has grown and reached adulthood it can outcompete a newly arrived competitor and hence become resistant to competitive displacement, a factor thought to reduce the need for extreme colonization ability in the fugitive species [9, 60, 61]. These differences may explain the stronger effect we observe as a result of spatial variation in a single resource compared to the model of Gross [48] in which dispersal is implicit. In addition, allowing multiple colonies to inhabit one patch means that the dispersal-constrained strategy (DCF) is more likely to disperse within its natal patch than to a new patch, and thus more likely to compete with its parent and/or sibs [64]. This will lead to an increase in competition for DCF relative to ICF with decreasing habitat saturation (Additional file 3; see also [44]) which may help explain the ability of ICF to survive in harsher conditions than DCF (i.e., low resources and high disturbance rate). While offspring optimality models of plants often predict the opposite pattern because larger offspring can have a higher tolerance to environmental stress and thus higher establishment success [23, 47, 65], our simulations indicate that, even though our DCF colonies have a higher establishment success (i.e., a lower dispersal mortality), this is not necessarily translated into broad-scale dominance because of the countering influence of kin-competition arising from limited dispersal.

While the CCTO plays a large role in the maintenance of ICF-DCF coexistence, coexistence in spatially structured landscapes can be enhanced by mechanisms such as mass effects [i.e., effects of migration; 66] or niche differences [i.e., ‘species sorting’; 59]. Our contrast of simulations conducted in uniform and harlequin environments support these predictions, as we found a broadening of coexistence conditions in six of seven tested parameters in harlequin environments (and no change in the other parameter). In harlequin environments, DCF benefits from higher resource availability on good patches where it can competitively exclude ICF. However, ICF also gains further colonization opportunities as colonies die out in bad patches. This aligns with previous models of spatially structured landscapes [48], though our explicit treatment of dispersal additionally indicates that expansion by DCF is inhibited by discontinuity of good habitat. The introduction of environmental heterogeneity therefore generates a landscape-structured fitness asymmetry overlay to the CCTO, magnifying refuges for both strategies and extending coexistence conditions. Our inclusion of simulations of different degrees of habitat aggregation highlight the influence of environmental heterogeneity (Fig. 5), as DCF was increasingly found on good patches with increasing aggregation while ICF was always randomly distributed. Increasing aggregation increases the chances that dispersing DCF colonies (i.e., those produced on good patches) will encounter like patches. At the same time, increasing aggregation restricts access to poor patches because DCF has limited capacity to bridge spans of bad patches to reach distant good habitat. This is because bad patches severely limit reproduction and can become sinks under competition conditions (which are more likely to arise for DCF because of limited dispersal). Thus, with increasing aggregation, DCF can access and monopolize more of the good habitat but less of the poor habitat. ICF, on the other hand is unaffected by habitat aggregation as its dispersal is (at least in this context) global. These findings support studies suggesting that landscape effects can act synergistically with the CCTO to facilitate coexistence [9, 48]. They also highlight the role of dispersal from good to bad patches as an important factor underlying the coexistence of strategies, stressing the importance of mass effects for the overall structure of the metacommunity [59, 66].

Many studies have considered the evolution of dispersal in a competition context and, while the size of this body of work puts a detailed review beyond the scope of this article [see for example 16], it is worth drawing attention to some parallels. For instance, situations in which we find a mutual invasibility of the two strategies are akin to works predicting that a polymorphism in dispersal (i.e., the coexistence of high and low dispersal strategies) can arise from spatial [e.g., 44, 45] or temporal [46] variation in the environment. Our observation that coexistence is facilitated in harlequin metacommunities is therefore in line with previous studies showing how spatio-temporal variation in the environment can allow the coexistence of contrasting dispersal strategies. Finally, whereas we consider DCF and ICF as competitor and colonizer strategies, Bolker and Pacala [67] show that, given a resident strategy with high dispersal (here, ICF), spatial segregation can be expected (in terms of density, and hence of competition intensity), and this can favour an ‘exploiter’ strategy which disperses locally and pre-empts space and resources by having high fecundity. As pointed out in Eqs. (1–6), differences in fecundity largely determine the conditions under which DCF should be expected to dominate the metacommunity. Thus, DCF, as a strategy relying on local dispersal and high fecundity, could be considered an ‘exploiter’ strategy [sensu 67]. However, Bolker and Pacala [67] assume that the ability to effectively exploit resources (competitive ability) is associated with the ability to tolerate low resources. This could not be tested in our model as resources are replenished, but seems to contradict our findings that DCF does poorly in low resource environments because of the limiting effects of kin competition. Furthermore, ‘exploiter’ strategies are by definition out-competed by competitive resident strategies, though as we consider only two reproductive strategies in our model this possibility remains to be resolved.

The CCTO has previously been implicated as an important mechanism in structuring ant communities [68], and our simulations indicate that a CCTO can support coexistence of ant species employing different reproductive strategies in a wide range of conditions. However, ant species can differ markedly in a variety of life-history traits which may additionally facilitate coexistence, making it very difficult to infer a CCTO effect in nature [though see 39 for an example among ICF ants]. This differs from our model in which we consider an all-else-equal scenario. Nonetheless, our model and results relate well to numerous cases of intraspecific polymorphism in reproductive strategies in ants [see 25, 69], and CCTOs may be important in sustaining such polymorphisms. For example, Heinze [41] found that DCF colonies of *Temnotothorax* (formerly *Leptothorax*) sp. A. were more common on isolated patches of good habitat (analogous to our aggregated landscapes) than in uniformly heterogeneous landscapes (analogous to our random landscapes), where the ICF form of the same species predominated. Similarly, Molet et al. [70] showed that the proportion of DCF colonies increased with latitude in an Australian *Rhytidoponera* species complex, and suggested this was linked with environmental quality. In addition, whereas our model compares two alternative and fixed strategies, some species have flexible strategies and may adaptively employ one or the other depending on environmental context, for example using DCF in times of environmental stress [71]. These species are thus able to exploit the advantages of both strategies and this may be more advantageous than either fixed strategy in some environments. Variation in life-history traits between sympatric ant species can be expected to further broaden coexistence conditions beyond those we demonstrate. Our findings are largely in line with previous predictions for environments favouring DCF or ICF [40–43]. We show that DCF is favoured by spatial and temporal homogeneity and factors favouring competitive ability, whereas ICF benefits from factors increasing colonization opportunities such as increased disturbance. In addition, ICF can persist in harsh landscapes because its high dispersal allows it to avoid kin-competition, which is likely to be an important constraint for DCF. However, DCF and ICF strategies represent extremes of the CCTO, and while the available evidence suggests these strategies are predominant, ants are likely to employ a far greater diversity of strategies than the examples used here. For instance, recent studies indicate that DCF is not limited to division into two, but instead can lead to the production of multiple new colonies of different size [25, 50, 72]. Intermediate strategies could be envisioned that combine beneficial traits of each strategy, particularly in other social hymenoptera in which workers can fly [25]. Future models should assess the viability of a range of intermediate strategies, and also incorporate the potential for evolution of strategies and genetic exchange between different modes of reproduction to model ‘mixed-strategist’ species. This would allow us to explore whether conditions identified here as favouring coexistence lead to stabilising selection (intermediate strategies) or disruptive selection (coexistence of two markedly different strategies). Finally, unlike assumed in our model, some species of ants are not sessile but frequently relocate their nests [reviewed in 73] to escape predators [74], find a better nesting sites [75] or track resources, as exemplified by the nomadic behavioural of army ants [37]. As emigrations can increase effective dispersal range, this could reduce the influence of kin competition on DCF.

CCTOs have been documented in a wide range of organisms (e.g., bacterial communities [76], parasitic trematodes [77], ciliates [78], beetles [79] and fish [80]), but most extensively in plants [3, 5, 9, 13]. Plant communities have strong parallels with those of many ants in being sessile following natal dispersal and in many cases having an offspring size-dispersal trade-off that is also linked with competitive ability. Reproduction in plants can further resemble ants with polymorphic reproductive strategies, as some plant species can disperse though both vegetative propagation and seeds [81]. Small seeds are analogous to ICF as they are cheaper to produce and can thus be produced in greater numbers while also having higher dispersal, and similarly suffer from lower competitive ability and lower establishment success [3, 9, 23]. Studies of other taxa align with our findings where dispersal is clearly important: Morrongiello et al. [82] report that an Australian freshwater fish invests in smaller, more numerous offspring where habitat is fragmented, but fewer, larger offspring where it is continuous, and attribute this to the importance of a colonization advantage to small offspring. Future studies should investigate the influence of a wider range of polymorphic and skewed reproductive strategies in different environments using models parameterized by ants and other organisms.

## Conclusions

We use a combined agent-based simulation and metacommunity modelling approach to demonstrate that the competition–colonisation trade-off can in many cases explain coexistence of different reproductive strategies and this coexistence is robust to a broad range of life-history traits and environmental conditions. Our novel spatially explicit approach allows us to disentangle spatial components of the coexistence mechanism from those related to more traditional competition–colonisation models, and highlights the importance of dispersal in facilitating conditions of coexistence in spatially structured environments and constraining the success of low-dispersers through kin-competition. These findings will contribute to our understanding of the links between different reproductive strategies in ants in different environments, and may help explain the evolution and maintenance of the remarkable diversity of reproductive strategies in the Formicidae.

## Additional files

10.1186/s12898-016-0058-z This file contains a detailed description of the model components and function following the format suggested by Railsback and Grimm [52].
10.1186/s12898-016-0058-z This file comprises the Netlogo model.
10.1186/s12898-016-0058-z This file includes additional figures summarising results from simulations, including summaries of the influence of each of the life-history and environmental parameters over the entire parameter ranges tested.

## Abbreviations

DCF: dependent colony foundation (also known as fission)
ICF: independent colony foundation
CCTO: competition–colonisation trade-off

## References

[1] Smith CC, Fretwell SD (1974). Optimal balance between size and number of offspring. Am Nat.

[2] Stearns SC (1992). The evolution of life histories.

[3] Geritz SAH, Van Der Meijden E, Metz JAJ (1999). Evolutionary dynamics of seed size and seedling competitive ability. Theor Popul Biol.

[4] Allen RM, Buckley YM, Marshall DJ (2008). Offspring size plasticity in response to intraspecific competition: an adaptive maternal effect across life-history stages. Am Nat.

[5] Moles AT, Westoby M (2004). Seedling survival and seed size: a synthesis of the literature. J Ecol.

[6] Lloyd DG (1987). Selection of offspring size at independence and other size-versus-number strategies. Am Nat..

[7] Parker GA, Begon M (1986). Optimal egg size and clutch size—effects of environment and maternal phenotype. Am Nat.

[8] Marshall DJ, Keough MJ (2008). The evolutionary ecology of offspring size in marine invertebrates. Adv Mar Biol.

[9] Coomes DA, Grubb PJ (2003). Colonization, tolerance, competition and seed-size variation within functional groups. Trends Ecol Evol.

[10] Burgess SC, Bode M, Marshall DJ (2013). Costs of dispersal alter optimal offspring size in patchy habitats: combining theory and data for a marine invertebrate. Funct Ecol.

[11] Bonte D, Van Dyck H, Bullock JM, Coulon A, Delgado M, Gibbs M (2012). Costs of dispersal. Biol Rev.

[12] Geritz SAH (1995). Evolutionarily stable seed polymorphism and small-scale spatial variation in seedling density. Am Nat.

[13] Kisdi E, Geritz SAH (2003). On the coexistance of perennial plants by the competition–colonization trade-off. Am Nat.

[14] Crean AJ, Marshall DJ (2009). Coping with environmental uncertainty: dynamic bet hedging as a maternal effect. Philos Trans R Soc Lond B.

[15] Einum S, Fleming IA (2004). Environmental unpredictability and offspring size: conservative versus diversified bet-hedging. Evol Ecol Res.

[16] Levin SA, Muller-Landau HC (2000). The evolution of dispersal and seed size in plant communities. Evol Ecol Res.

[17] Starrfelt J, Kokko H, Clobert J, Baguette M, Benton TG, Bullock JM (2012). The theory of dispersal under multiple influences. Dispersal ecology and evolution.

[18] Aldermane J, McCollin D, Hinsley SA, E. BP, Picton P, Crockett R. Modelling the effects of dispersal and landscape configeration on population distribution and viability in a fragmented habitat. Landsc Ecol. 2005;20:857-70.

[19] Tilman D, May RM, Lehman CL, Nowak MA (1994). Habitat destruction and the extinction debt. Nature.

[20] Bonte D, Borrre JV, Lens L, Maelfait J-P (2006). Geographical variation in wolf-spider dispersal behaviour is relatied to landscape structure. Anim Behav.

[21] Cheptou PO, Carrue O, Rouifed S, Cantarel A (2008). Rapid evolution of seed dispersal in an urban environment in the weed *Crepis sancta*. Proc Natl Acad Sci USA.

[22] Marshall DJ, Cook CN, Emlet RB (2006). Offspring size effects mediate competitive interactions in a colonial marine invertebrate. Ecology.

[23] Muller-Landau HC (2010). The tolerance–fecundity trade-off and the maintenance of diversity in seed size. Proc Natl Acad Sci USA.

[24] Rollinson N, Hutchings JA (2013). Environmental quality predicts optimal egg size in the wild. Am Nat..

[25] Cronin AL, Molet M, Doums C, Monnin T, Peeters C (2013). Recurrent evolution of dependent colony foundation across eusocial insects. Annu Rev Entomol.

[26] Weppler T, Stoll P, Stöcklin J (2006). The relative importance of sexual and clonal reproduction for population growth in the long-lived alpine plant *Geum reptans*. J Ecol.

[27] Van Kleunen M, Fischer M, Schmid B (2001). Effects of intraspecific competition on size variation and reproductive allocation in a clonal plant. Oikos.

[28] Douhovnikoff V, Cheng AM, Dodd RS (2004). Incidence, size and spatial structure of clones in second-growth stands of coast redwood *Sequoia sempervirens* (Cupressaceae). Am J Bot.

[29] Burnett AL (1973). The biology of hydra.

[30] Foster NL, Baums IB, Mumby PJ (2007). Sexual vs. asexual reproduction in an ecosystem engineer: the massive coral *Montastraea annularis*. J Anim Ecol.

[31] McNutt JW (1996). Sex-biased dispersal in African widl dogs, *Lycaon pictus*. Anim Behav.

[32] de Casas RR, Willis CG, Donohue K, Clobert J, Baguette M, Benton TG, Bullock JM (2012). Plant dispersal phenotypes: a seed perspective of maternal habitat selection. Dispersal ecology and evolution.

[33] Tilman D (1994). Competition and biodiversity in spatially structured habitats. Ecology.

[34] Hastings A (1980). Disturbance, coexistence, history and competition for space. Theor Popul Biol.

[35] Tschinkel WR (2006). The fire ants.

[36] Peeters C, Molet M, Lach L, Parr C, Abbott K (2010). Colonial reproduction and life histories. Ant ecology.

[37] Gotwald WHJ (1995). Army Ants: The Biology of Social Predation.

[38] Parr CL, Gibb H, Lach L, Parr C, Abbott K (2010). Competition and the role of dominant ants. Ant ecology.

[39] Stanton ML, Palmer TM, Young TP (2002). Competition–colonization trade-offs in a guild of African acacia-ants. Ecol Monogr.

[40] Bourke AFG, Franks NR (1995). Social evolution in ants.

[41] Heinze J (1993). Habitat structure, dispersal strategies and queen number in two boreal *Leptothorax* ants. Oecologia.

[42] Bourke AFG, Heinze J (1994). The ecology of communal breeding: the case of multiple-queen leptothoracine ants. Philos Trans R Soc Lond B.

[43] Heinze J, Tsuji K (1995). Ant reproductive strategies. Res Popul Ecol.

[44] Massol F, Duputié A, David P, Jarne P (2011). Asymmetric patch size distribution leads to disruptive selection on dispersal. Evolution.

[45] Parvinen K (2002). Evolutionary branching of dispersal strategies. J Math Biol.

[46] Mathias A, Kisdi E, Olivieri I (2001). Divergent evolution of dispersal in a heterogeneous landscape. Evolution.

[47] Adler PB, Fajardo A, Kleinhesselink AR, Kraft NJB (2013). Trait-based tests of coexistence mechanisms. Ecol Lett.

[48] Gross K (2008). Fusing spatial resource heterogeneity with a competition–colonization trade-off in model communities. Theor Ecol.

[49] Wilensky U (1999). NetLogo.

[50] Chéron B, Cronin AL, Doums C, Fédérici P, Haussy C, Tirard C (2011). Unequal resource allocation among colonies produced by fission in the ant *Cataglyphis cursor*. Ecology.

[51] Rangel J, Seeley TD (2012). Colony fissioning in honey bees: size and significance of the swarm fraction. Insectes Soc.

[52] Railsback SF, Grimm V (2012). Agent-based and individual-based modelling: a practical introduction.

[53] Leibold MA, Loeuille N (2015). Species sorting and patch dynamics in harlequin metacommunities: influences on the environmental and spatial regulation of community composition. Ecology.

[54] Horn HS, MacArthur RH (1972). Competition among fugitive specie in a harlequin environment. Ecology.

[55] Saupe D, Petigen HO, Saupe D (1988). Algorithms for random fractals. The science of fractal images.

[56] King AW, With KA (2002). Dispersal success on spatially structured landscapes: when do spatial pattern and dispersal behavior really matter?. Ecol Model.

[57] Peeters C, Ito F (2001). Colony dispersal and the evolution of queen morphology in social Hymenoptera. Annu Rev Entomol.

[58] Tilman D (1986). Nitrogen-limited growth in plants from different successional stages. Ecology.

[59] Leibold MA, Holyoak M, Mouquet N, Amarasekare P, Chase JM, Hoopes MF (2004). The metacommunity concept: a framework for multi-scale community ecology. Ecol Lett.

[60] Higgins SI, Cain ML (2002). Spatially realistic plant metapopulation models and the colonization–competition trade-off. J Ecol.

[61] Calcagno V, Mouquet N, Jarne P, David P (2006). Coexistence in a metacommunity: the competition–colonization trade-off is not dead. Ecol Lett.

[62] Yu DW, Wilson HB, Frederickson ME, Palomino W, De La Colina R, Edwards DP (2004). Experimental demonstration of species coexistence enabled by dispersal limitation. J Anim Ecol.

[63] Aiken CM, Navarrete SA (2014). Coexistence of competitors in marine metacommunities: environmental variability, edge effects, and the dispersal niche. Ecology.

[64] Hamilton WD, May RM (1977). Dispersal in stable habitats. Nature.

[65] Einum S, Flemming IA (2000). Highly fecund mothers sacrifice offspring survival to maximize fitness. Nature.

[66] Mouquet N, Loreau M (2003). Community patterns in source-sink metacommunities. Am Nat..

[67] Bolker BM, Pacala SW (1999). Spatial moment equations for plant competition: understanding spatial strategies and the advantage of short dispersal. Am Nat.

[68] Hölldobler B, Wilson EO (1990). The ants.

[69] Peeters C (2012). Convergent evolution of wingless reproductives across all subfamilies of ants, and sporadic loss of winged queens (Hymenoptera: Formicidae). Myrmecol News.

[70] Molet M, van Baalen M, Peeters C (2008). Shift in colonial reproductive strategy associated with a tropical-temperate gradient in *Rhytidoponera* ants. Am Nat.

[71] Briese DT (1983). Different modes of reproductive behaviour (including a description of colony fission) in a species of *Chelaner* (Hymenoptera: Formicidae). Insectes Soc.

[72] Cronin AL, Fédérici P, Doums C, Monnin T (2012). The influence of intraspecific competition on resource allocation during dependent colony foundation in a social insect. Oecologia.

[73] McGlynn TP (2012). The ecology of nest movement in social insects. Annu Rev Entomol.

[74] McGlynn TP, Carr RA, Carson JH, Buma J (2004). Frequent nest relocation in the ant *Aphaenogaster araneoides*: resources, competition, and natural enemies. Oikos.

[75] Dornhaus A, Franks NR, Hawkins RM, Shere HNS (2004). Ants move to improve: colonies of *Leptothorax albipennis* emigrate whenever they find a superior nest site. Anim Behav.

[76] Livingston G, Matias M, Calcagno V, Barbera C, Combe M, Leibold MA (2012). Competition–colonization dynamics in experimental bacterial metacommunities. Nat Commun.

[77] Soldánová M, Kostadinova A (2011). Rapid colonisation of *Lymnaea stagnalis* by larval trematodes in eutrophic ponds in central Europe. Int J Parasitol.

[78] Limberger R, Wickham SA (2011). Competition–colonization trade-offs in a ciliate model community. Oecologia.

[79] Kadowaki K, Leschen RAB, Beggs JR (2011). Competition–colonization dynamics of spore-feeding beetles on the long-lived bracket fungi *Ganoderma* in New Zealand native forest. Oikos.

[80] Pfister CA (2006). Concordance between short-term experiments and long-term censuses in tide pool fishes. Ecology.

[81] Winkler E, Fischer M (2002). The role of vegetative spead and seed dispersal for optimal life histories in plants: a simulation study. Evol Ecol.

[82] Morrongiello JR, Bond NR, Crook DA, Wong BBM (2012). Spatial variation in egg size and egg number reflects trade-offs in bet-hedging in a freshwater fish. J Anim Ecol.

