# Supplementary material for: Strategies of offspring investment and dispersal in a spatially structured environment: a theoretical study using ants
Source: BMC Ecol. 2016 Feb 5;16:4. doi: 10.1186/s12898-016-0058-z (PMC4743417; doi:10.1186/s12898-016-0058-z)
Supplement: Supplementary file 1 — 10.1186/s12898-016-0058-z This file contains a detailed description of the model components and function following the format suggested by Railsback and Grimm [52]. [file 12898_2016_58_MOESM1_ESM.docx]

**Strategies of offspring investment and dispersal in a spatially structured environment: a theoretical study using ants**

Adam L. Cronin, Nicolas Loeuille and Thibaud Monnin

**Additional file 1: Model ODD (Overview, Design concepts and Details)**

**ODD – June 2015**

**Netlogo model of competition and colonisation in a spatially explicit environment**

Associated publication: Strategies of offspring investment and dispersal in a spatially structured environment: a theoretical study using ants, by A. L. Cronin, N. Loeuille & T. Monnin

**OVERVIEW**

1. **Purpose**

The model compares the effectiveness of different modes of reproduction and dispersal (here colony foundation in ants) under conditions of varied environmental and life-history parameters. It seeks to show under which conditions one strategy is favoured over the other and under which conditions strategies can coexist. This model permits modelling of two reproductive strategies in an environment consisting of variable habitat types. The characteristics, prevalence and association (aggregation) of the habitat types can be varied, permitting modelling of spatial and temporal variation. Agents are defined by several variable life-history traits that are shared between strategies and several reproductive traits unique to each strategy. We designed the model to be as generic as possible to enable modelling a wide range of organisms and environmental conditions, though we focus here specifically on the ecology of different reproductive strategies in ants. Specific strategies to be tested model ICF (independent colony foundation) and DCF (dependent colony foundation) in ants: under ICF, reproductive investment in each offspring is minimal and dispersal is maximal, whereas under DCF reproductive investment in each unit is maximal while dispersal is minimal ([see also: Peeters and Molet 2010](#_ENREF_5); [Cronin et al. 2013](#_ENREF_1)).

1. **Entities, state variable, and scales**

The model has two components: patches and individuals (agents). Agents can represent any living organism with variable reproductive investment.

**Agents**

Agents are defined by life-history parameters that are universal to all agents and other parameters specific to each of the reproductive strategies. Agents are static (immobile) and may occur anywhere in a patch. Agents only move when new agents are generated (reproduction), at which time they disperse a variable distance depending on the reproductive strategy. Resources available to each agent are represented by agent size. Agents accumulate resources from their patch which are allocated to maintenance, growth and reproduction. Agents die at a set age. Since resources and individuals can freely be converted into one another, colony size can be viewed either as the number of individuals making up a colony or as the quantity of resources it contains. At each step of the simulations, we recorded for each of the 961 patches the total number of agents of each strategy on each patch class (i.e. number of ICF/DCF colonies on good/bad patches). Agents were divided arbitrarily into ‘large’ (defined as those with size exceeding 75% of maturity threshold) and ‘small’ to broadly investigate patterns of agent maturity. We also recorded the summed size of agents on patches of each class to quantify distribution with respect to reproductive strategy and maturity.

**Patches**

The environment is a continuous region (i.e. wrapping toroid) comprising 31x31 square patches, each of which generates resources and can be occupied by any number of agents. Patches generate resources, which can be harvested by agents in the patch. Patches may vary in quality, which influences the resources available. Patch quality may vary spatially depending on model settings. Patches can thus be seen as territories whose resources are shared between the resident colonies. We use two main forms of environment: ‘uniform’ and ‘harlequin’ landscapes ([Horn and MacArthur 1972](#_ENREF_2); [Leibold and Loeuille 2015](#_ENREF_4)) comprising patches of two classes, ‘bad’ and ‘good’, with bad patches defined as of having half of the quality of good patches. Harlequin landscapes are constructed of good and bad patches in user-defined proportions whereas in uniform landscapes, all patches are set to an intermediate level between these extremes (‘medium’ patches) which ensures that the overall amount of resources available at the level of the landscape remains constant in both harlequin and uniform landscapes. Patch distribution in harlequin landscapes is either ‘random’ or ‘aggregated’. In ‘random’ landscapes, good and bad patches are randomly distributed. For aggregated habitats, fractal landscapes are generated using the midpoint-displacement method ([Saupe 1988](#_ENREF_6)) and Hurst exponents of 0, 0.5 and 1, to generate low, medium and high levels of ‘spatial contagion’ of like patches ([see King and With 2002](#_ENREF_3)).

Each step in the simulation represents one reproductive event i.e. one year. Simulations are run for 1000 steps as testing indicated this ensured an equilibrium state had been achieved. In addition, an ‘invasion’ option is included in which simulations are run for a preliminary user-set duration with a single strategy before a single individual of the alternative strategy is introduced. These simulations are run for 3000 steps to allow equilibrium to be achieved.

**Assumptions of the model**

We assume that: i) all agents are able to produce offspring once they grow above a threshold size; ii) there is no genetic exchange between DCF and ICF agents (i.e. they are effectively treated as reproducing asexually or as separate species). With respect to ant reproductive strategies we assume that variation in dispersal success between strategies eclipses that within strategies, and thus whereas individual dispersal (ICF) is high risk ([e.g. Tschinkel 2006](#_ENREF_7)), group dispersal (DCF) is low risk, regardless of the within-strategy offspring size. Finally, as we are interested in examining the influence of spatio-temporal status of the environment on strategy success, we make the simplifying assumption that there are no environmental feedbacks related to continued occupancy: patches are considered to be broadly stable over simulated time and neither accumulate resources nor degrade.

1. **Process overview and scheduling**

The landscape is initialised using user-set parameters for spatial structure and habitat composition. It is populated with agents with random distribution and user-defined characteristics. The annual cycle consists of six phases (in bold below), details of which are given in the section on sub-models (below). A flow-diagram of the model is presented in Figure 1 (below). The year beings when patches **Produce Resources**. Agents compete with other agents in their patch to **Collect Resources** and then pay **Maintenance cost**. Agents of sufficient size then **Reproduce**; new offspring produced then **Disperse** and those that survive are added to the list of active agents. Finally, at the end of each annual phase, age is incremented and agents **Live or Die**; those which reach a size of 0 or less (through competition and maintenance), exceed the maximum age, or are the victim of stochastic mortality (disturbance events), are removed.

**DESIGN CONCEPTS**

1. **Design concepts**
   1. **Basic principles**

The basic principle addressed is the ubiquitous trade-off between size and number of offspring (propagules), with the added complexity of dispersal in a spatially structured environment. The model identifies conditions under which one strategy prevails over or co-occurs with another.

- 1. **Emergence**

The final number of agents at the termination of the model is a product of individual reproduction which itself is determined by interactions between individuals and spatio-temporal distribution of resources. The final number and spatial distribution of agents is thus an emergent property of the interaction between agents and their environment.

- 1. **Adaptation**

There is no adaptation in the model.

- 1. **Sensing**

Dispersing agents are able to determine characteristics of possible dispersal sites under some settings and make choices among possible sites accordingly. Under default settings there is no sensing and dispersal is random.

- 1. **Interaction**

Agents interact indirectly within a patch. All agents present receive a share of available resources depending on i) their relative competitive ability (a function of size) and ii) their foraging ability (also a function of size but independent of other colonies). Each of these factors defines a maximum resource value, and agents receive the lowest one of these each turn.

- 1. **Stochasticity**

When initialising the model, the initial resources of patches and the location of colonies are randomly determined. Agents are processed in a random order each turn. Dispersal of agents is random when not selective (see 4.4 Sensing).

Environmental stochasticity is represented by a fixed rate of annual variation in resource input to patches of each class (± 10%), and an adjustable proportion of random patches on which all agents are killed (disturbance). Landscape structure in landscapes of mixed patch types is randomly determined in each case, with either completely stochastic distribution of patches or using a fractal algorithm based on stochastic seeds.

- 1. **Observation**

At the end of each year, the total number of agents of each reproductive strategy is recorded. In addition, the number of agents of each strategy on each class of habitat patch is recorded, as is the number of ‘large’ agents (those that have attained a size of at least 75% of reproductive size) on each class of habitat patch. In comparative analyses, only the final population size of each of these (i.e. at step 1000, or step 3000 for invasion scenarios) is used.

**DETAILS**

1. **Initialization**

Spatial structure of the landscape and patch resources are set when the model starts. Agents are randomly distributed, with a range of universal (life-history) and strategy specific (reproductive) parameters (see Table 1 in main text). A reference model was run to define a base-line using parameter settings considered to represent neutral and/or realistic values (Table 1 in main text). The influence of the modification of individual parameters was then examined while leaving all other parameters at their reference values using Behaviour Space in NetLogo 5.1.

1. **Sub-models**

Details of each phase of the model are described below. Numbers in each case refer to stages in the model flow diagram (Figure 1).

**
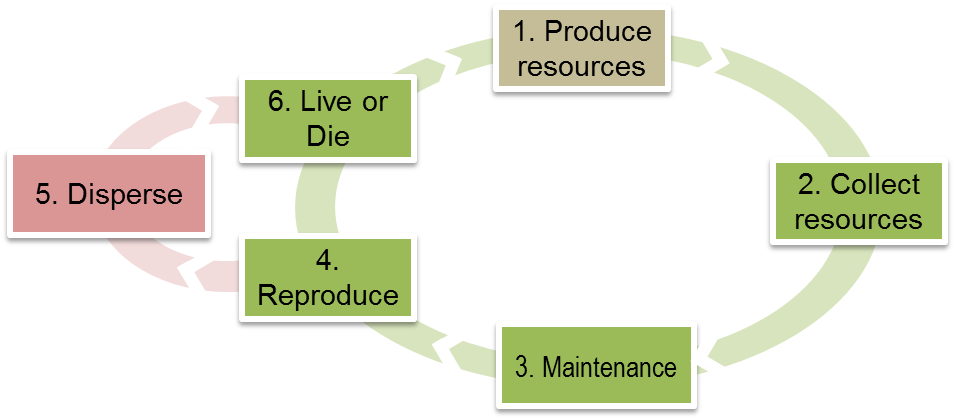
**

**Figure 1. Model flow diagram**

- **1. Produce resources:** Patches generate resources based on the base environmental resource level and patch quality. Class 1 patches are defined as of patch quality 1, whereas class 2 patches are a user-set proportion of this value. In each case resources in each patch are thus set to [base resource input × patch quality] and this is further modified by a stochastic component of ± 10%.
- **2. Collect resources:** colonies collect resources from their patch, according to available resources, agent size and competition. Each agent receives a share of the available resources equal to the lesser of i) their size relative to the size of all agents present (competition limit) and ii) a maximum possible resources collectable equal to their size multiplied by the maximum growth rate (size dependent limit). Resources thus gained are added to the agent’s reservoir (size).
- **3. Maintenance cost:** Colonies pay a cost of maintenance (a percentage of size) from their current resource store (size).
- **4. Reproduce:** Agents above a threshold size reproduce. Agents invest 0-100% of current resources (size) into new agents. The reproductive mode determines the number and size of new agents produced. ICF agents produce *n* new agents of size *x*, where *n* is the maximum number of agents of size *x* that can be produced. DCF agents produce one new agent of size equal to (parent size x reproductive investment). Invested resources are in both cases removed from the parent agent.
- **5. Disperse:** New agents disperse a user set range, either randomly or by selecting a patch in range using user set criteria. Three alternative modalities of dispersal within a set range are: (1) random dispersal; (2) dispersal to the patch with the smallest total competitive agent size (randomly chosen if two or more patches have equal size); (3) dispersal to the patch holding most resources. Dispersing agents have a user set chance of surviving dispersal depending on the reproductive strategy. Dispersers do not interact with colonies residing in patch(es) they cross.
- **6. Live or die:** Agents age each year, and are checked to see if they survive the turn, based on: i) if age exceeds set longevity, ii) if size reaches 0 from maintenance costs and lack of resource acquisition, or iii) random events (‘acts of god’) in which all agents on a user set proportion of patches are destroyed through disturbance events.

**References**

Cronin AL, Molet M, Doums C, Monnin T, Peeters C (2013) Recurrent evolution of dependent colony foundation across eusocial insects. Annu Rev Entomol 58:37-55

Horn HS, MacArthur RH (1972) Competition among fugitive specie in a harlequin environment. Ecology 53:749-752

King AW, With KA (2002) Dispersal success on spatially structured landscapes: when do spatial pattern and dispersal behavior really matter? Ecol Model 147:23-29

Leibold MA, Loeuille N (2015) Species sorting and patch dynamics in harlequin metacommunities: Influences on the envronmental and spatial regulation of community composition. Ecology XX:xxx-xxx

Peeters C, Molet M (2010) Colonial reproduction and life histories. In: Lach L, Parr C, Abbott K (eds) Ant Ecology. Oxford University Press, Oxford, UK, pp 159-176

Saupe D (1988) Algorithms for random fractals. In: Petigen HO, Saupe D (eds) The Science of Fractal Images. Springer, New York, pp 71-113

Tschinkel WR (2006) The Fire Ants. Harvard University Press
