# Supplementary material for: Strategies of offspring investment and dispersal in a spatially structured environment: a theoretical study using ants
Source: BMC Ecol. 2016 Feb 5;16:4. doi: 10.1186/s12898-016-0058-z (PMC4743417; doi:10.1186/s12898-016-0058-z)
Supplement: Supplementary file 3 — 10.1186/s12898-016-0058-z This file includes additional figures summarising results from simulations, including summaries of the influence of each of the life-history and environmental parameters over the entire parameter ranges tested. [file 12898_2016_58_MOESM3_ESM.docx]

**Strategies of offspring investment and dispersal in a spatially structured environment: a theoretical study using ants**

Adam L. Cronin, Nicolas Loeuille and Thibaud Monnin

**Additional File 3: Supplementary figures**

**
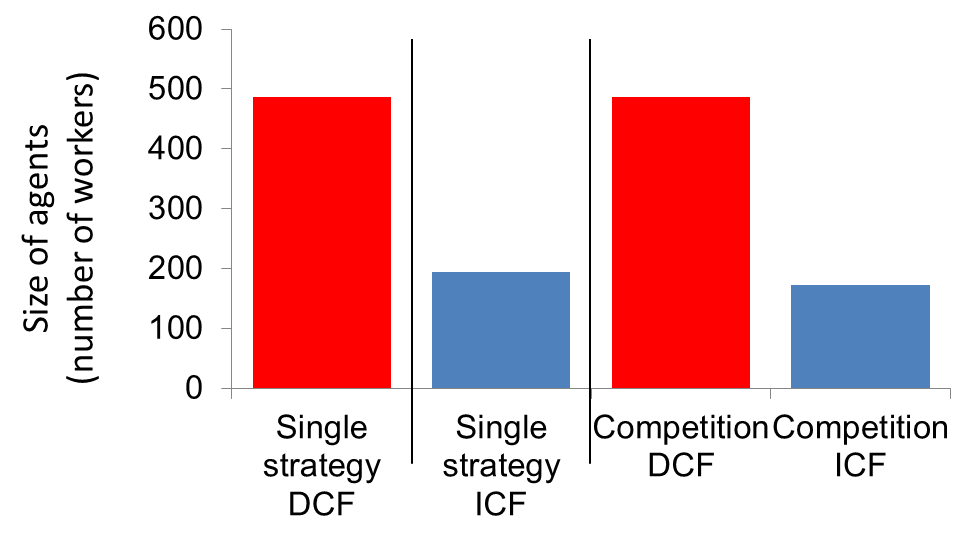
**

**Figure A1**. Mean size of agents under single-strategy and competition scenarios in uniform landscape. Red bars indicate DCF colonies and blue bars ICF colonies


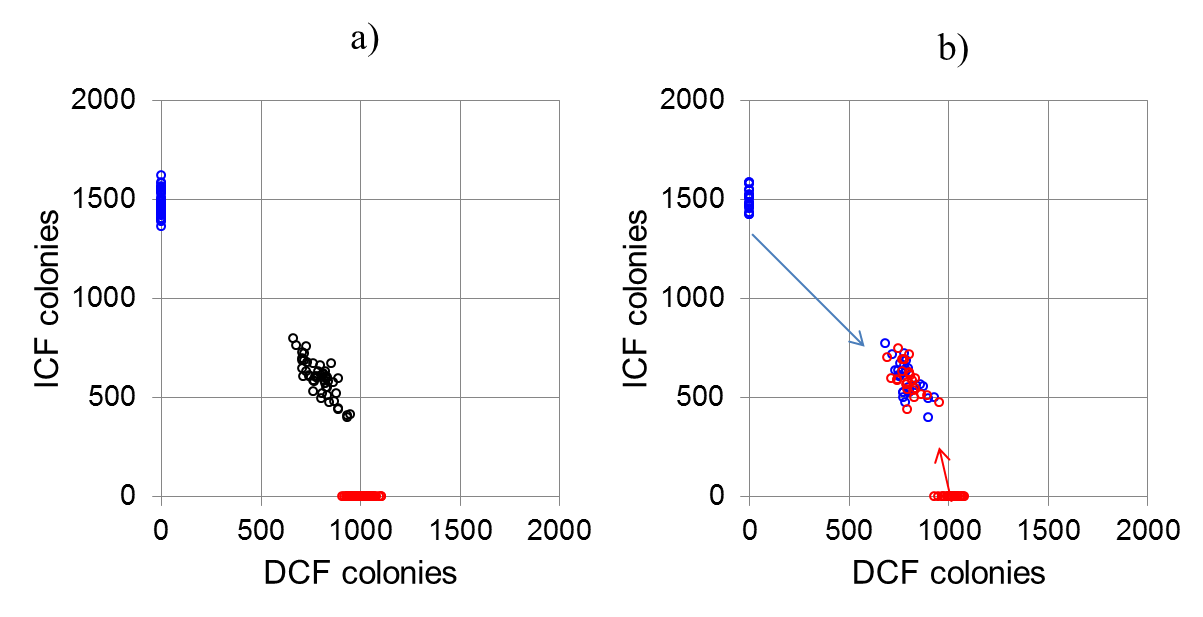


**Figure A2.** Outcomes of the three scenarios modelled using the reference parameters. (a) shows the number of colonies at the end of the “single-strategy” scenarios (with only DCF colonies = red dots; with only ICF = blue dots) and “competition” scenario (with both strategies, black dots). (b) shows the outcome of invasion (in red = ICF invading DCF; in blue = DCF invading ICF). When the invader failed the resident strategy produced as many colonies as when it was alone (compare dots on x- and y-axis with red and blue dots in (a), respectively). When the invader succeeded (arrows) each strategy produced as many colonies as under the “competition” scenario (compare dots on the centre of the plot with black dots in (a)).

**Figure A3 – Influence of model parameters on outcome of simulations in uniform landscapes**

Competition figures show the number of colonies in single-strategy (dots) and competition (solid) scenarios.

Invasion figures show the percentage of successful invasions (solid) and the percentage of invader colonies at the end (dots).

NOTE: in uniform environments the x-axis for resources reflects that for good patches, not medium patches


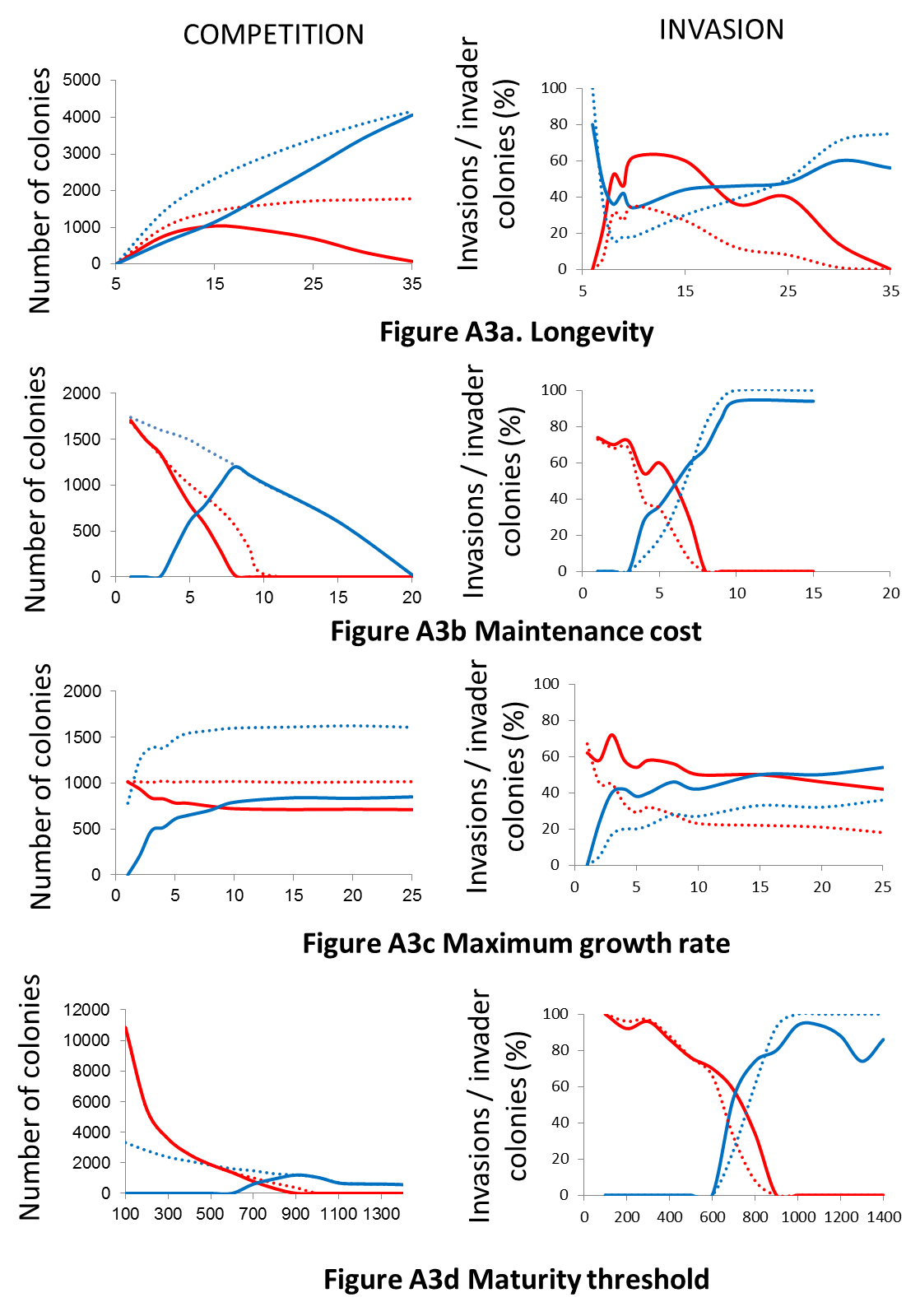


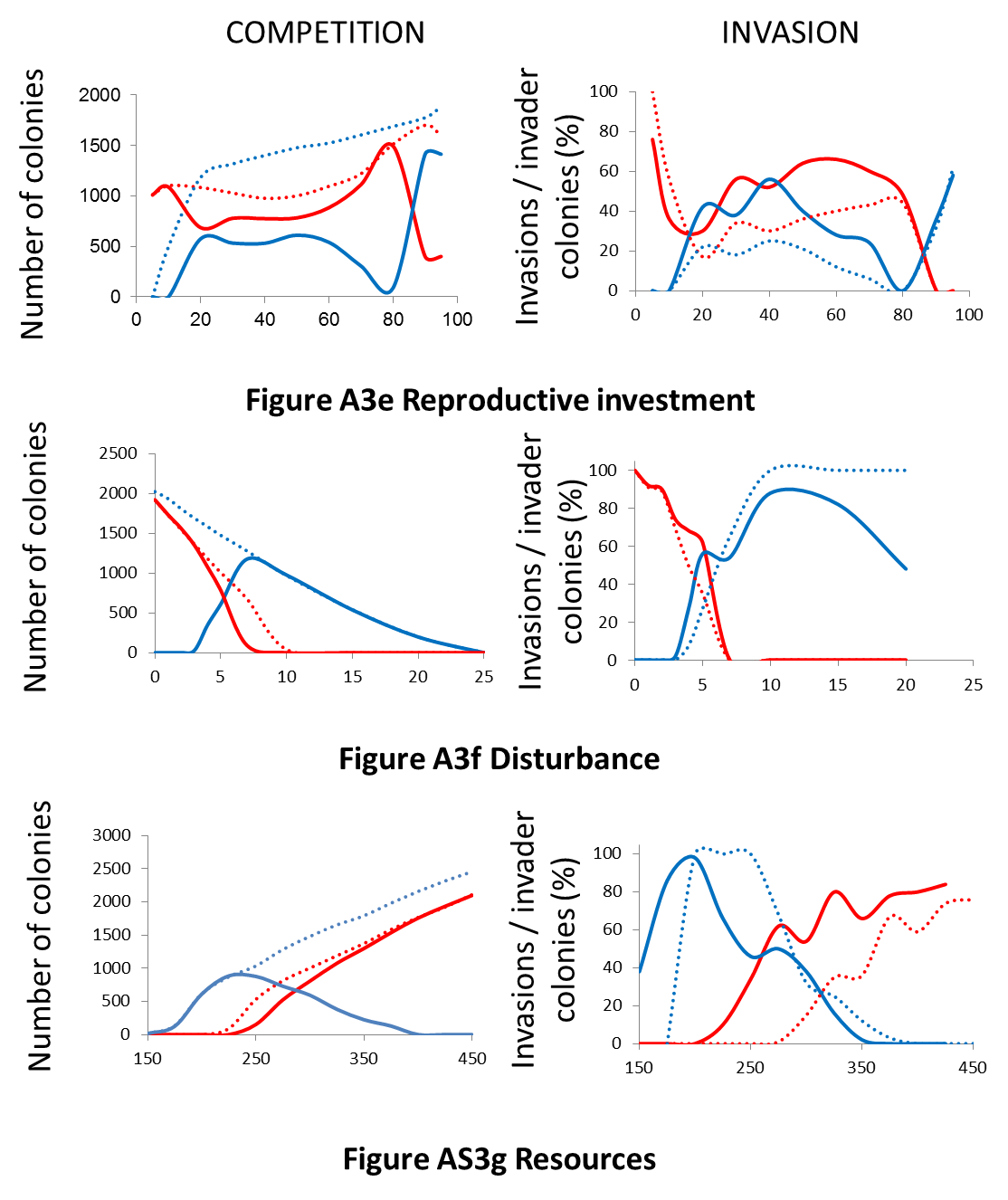


**Figure A4 – Influence of model parameters on outcome of simulations in harlequin landscapes**

Competition figures show the number of colonies in single-strategy (dots) and competition (solid) scenarios.

Invasion figures show the percentage of successful invasions (solid) and the percentage of invader colonies at the end (dots).


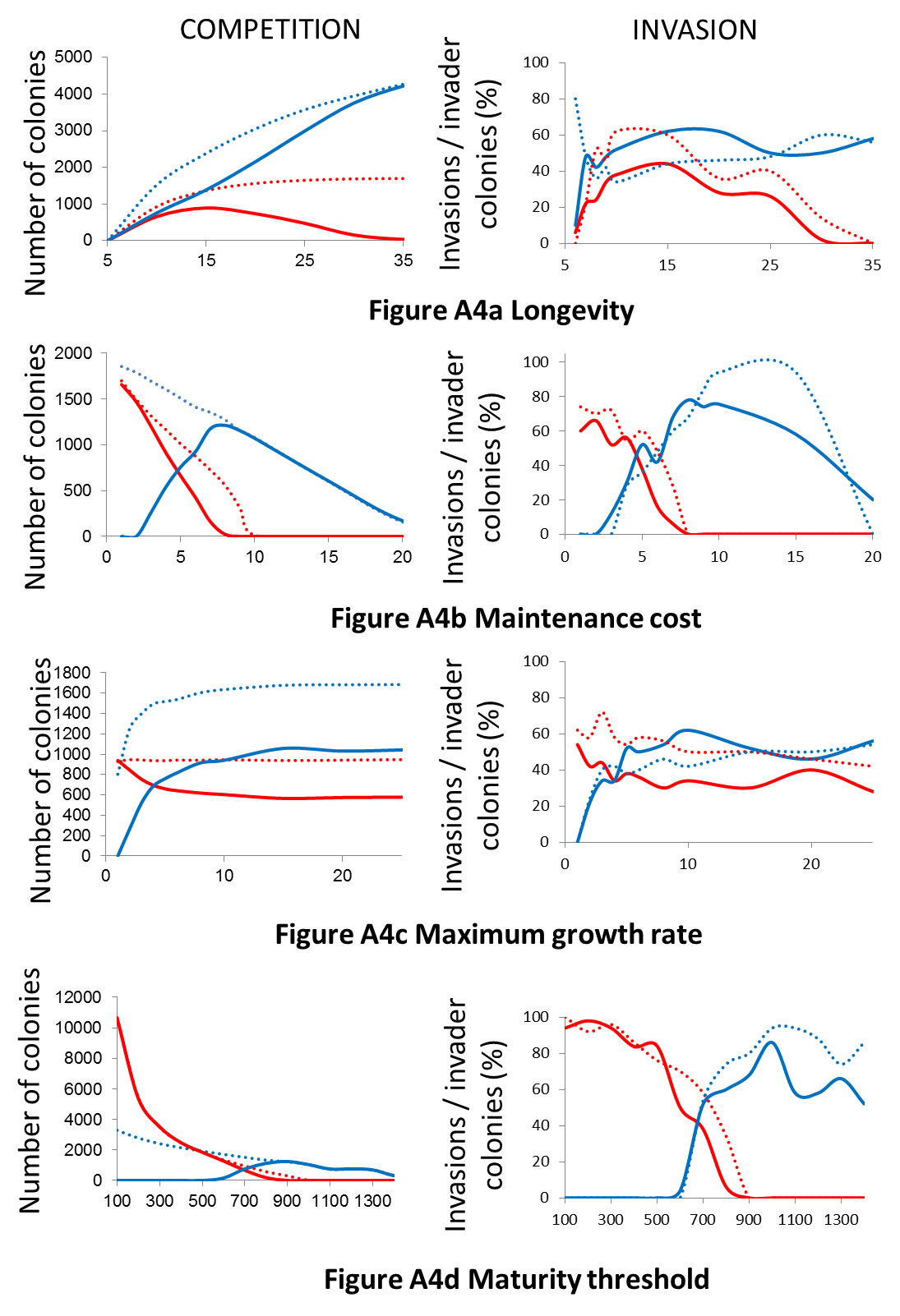


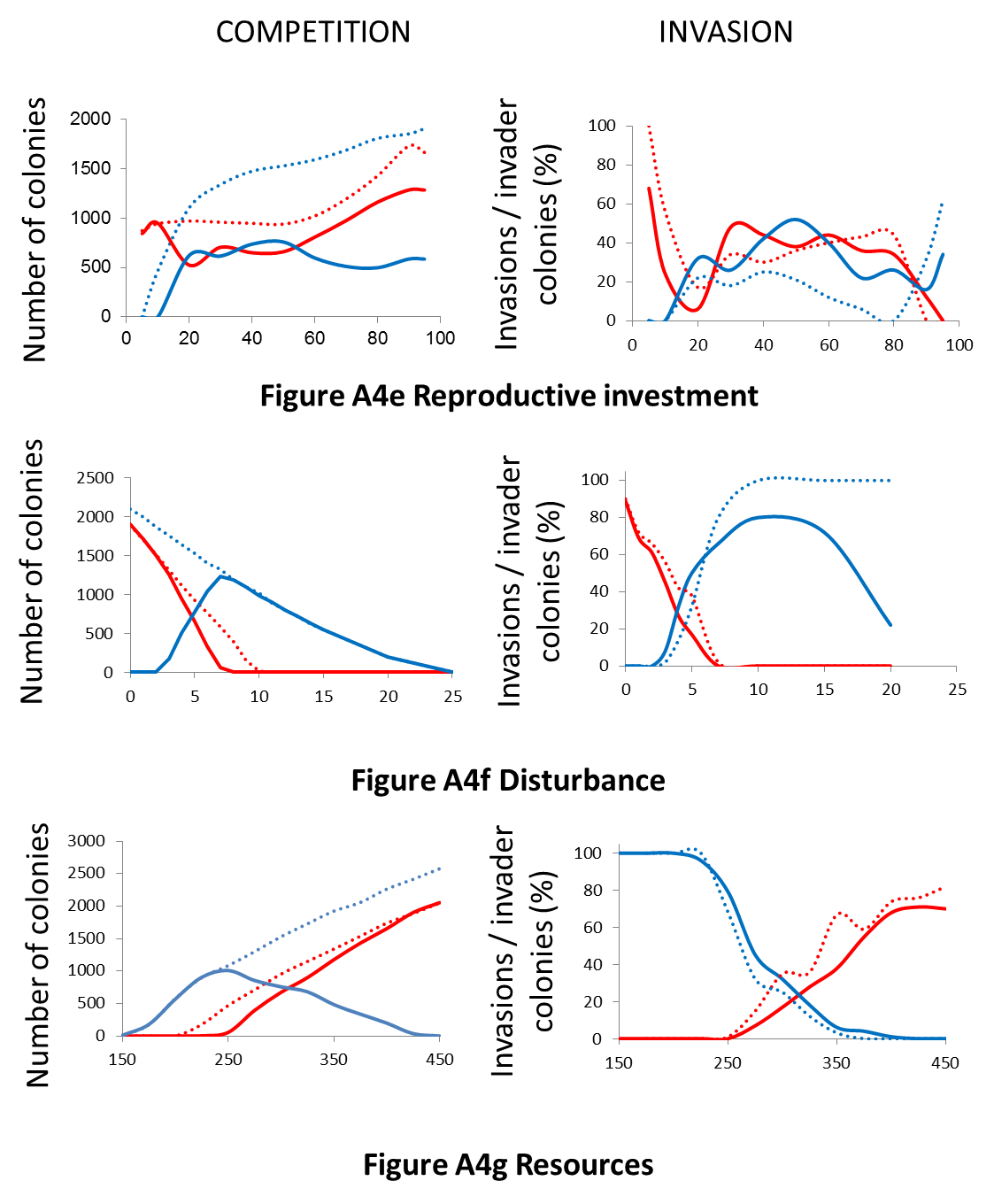


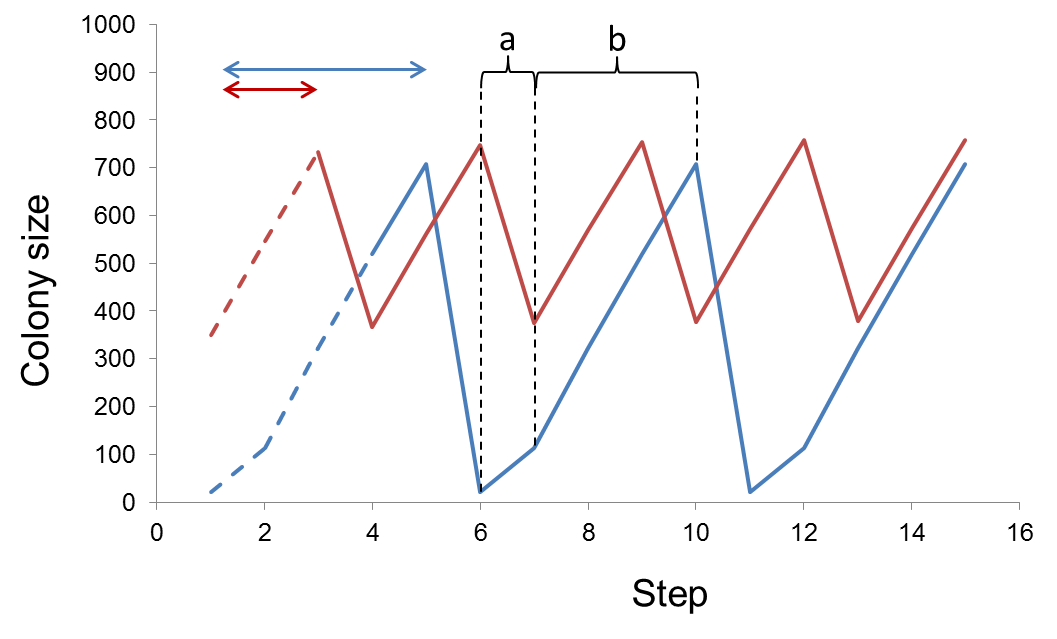


**Figure A5.** Growth rate and delay to maturity of DCF and ICF agents under a single-strategy scenario assuming a uniform environment and 5% maintenance costs. Agents of ICF (blue) and DCF (red) start as offspring (dotted lines) and begin reproducing as adults (solid lines). DCF offspring (350+ workers) grow rapidly to maturity (2 steps). ICF offspring start much smaller and are also influenced by size-related growth-rate limitations (a) which limits growth below that limited by resources until colonies are large enough to exploit all resources in a patch (b). Thus the time for ICF agents to reach maturity (4 steps), is increased relative to that of DCF agents (two-headed arrows).


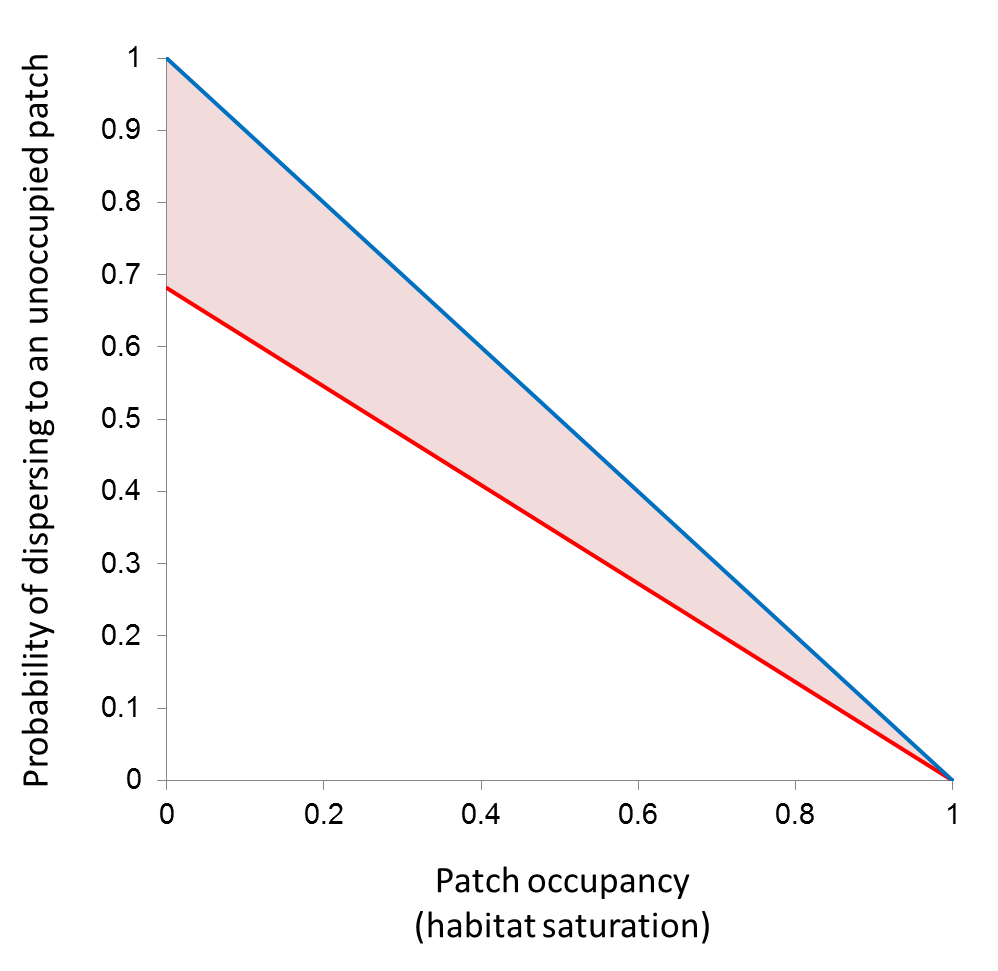


**Figure. A6**. Probability of dispersing to a vacant patch (*A*) as a function of habitat saturation for DCF (red) and ICF (blue). Calculated as probability an empty patch occurring within the area encompassed by dispersal range *(d*)*,* assuming the area of the home patch (*h*) is unavailable and all other patches are occupied at rate *p*, following:$A=\left( \pi d^{2}-h \right)\times(1-p)/\pi d^{2}$. Parameters used are: home patch 1x1, DCF dispersal range 1, and ICF dispersal range 30.
